# Morphological Modification and Analysis of ZnO Nanorods and Their Optical Properties and Polarization

**DOI:** 10.1155/2018/6545803

**Published:** 2018-11-05

**Authors:** Nandang Mufti, Siti Maryam, Anggun A. Fibriyanti, Robi Kurniawan, Abdulloh Fuad, Ahmad Taufiq, Sunaryono

**Affiliations:** ^1^Department of Physics, Faculty of Mathematics and Natural Sciences, Universitas Negeri Malang, Jl. Semarang 5, Malang, 65145 East Java, Indonesia; ^2^Centre of Advanced Materials for Renewable Energy, Universitas Negeri Malang, Jl. Semarang 5, Malang, 65145 East Java, Indonesia

## Abstract

We report on the effect of the morphological modification on optical properties and polarization of ZnO nanorods (NR). Here, the morphology and structure of the ZnO NR were modified by introducing different annealing temperatures. The increase of length and diameter and change in density of the ZnO NR were clearly observed by increasing the annealing temperature. We found that the samples show different oxygen vacancy (V_O_) and zinc interstitial (Zn_I_) concentrations. We suggest that the different concentrations of V_O_ and Zn_I_ are originated from morphological and structural modification. Our results reveal that optical absorption and polarization of ZnO NR could be enhanced by producing a high concentration of V_O_ and Zn_I_. The modification of V_O_ and Zn_I_ promotes a decrease in the band gap and coexistence of high optical absorption and polarization in our ZnO NR. Our findings would give a broad insight into the morphological modification and characterization technique on ZnO NR. The high optical and polarization characteristics of ZnO NR are potential for developing the high-performance nanogenerator device for multitype energy harvesting.

## 1. Introduction

In recent years, the increase in energy consumption is recorded in the industrial [1, 2] and global transportation [3, 4] sectors, mainly as a result of increased economic activity and mobility of people across the country. Among the various types of energy, fossil energy shows the greatest number of energy consumption to supply global electricity in the industrial and global transportation [3, 5]. The massive exploitation of fossil energy leads to the reduction in a large scale of fossil energy, and it takes a long time to be available again in nature, hundreds to thousands of years. In addition, the consumption of fossil energy is a major cause of environmental problems, such as global warming and air pollution [6–8]. Therefore, it is very important to find alternative fossil energy based on electricity from renewable materials, which is safe for the living environment and abundant in nature. Here, electrical energy can be generated from various resources available in the environment, such as kinetic energy (piezoelectric) [9, 10], heat energy (triboelectric) [11–14], and solar energy (photoelectric) [15–17]. In order to increase the number of energy harvesting and efficiency, hybrid nanogenerators have been developed, which can convert multienergy from nature [18–21]. However, the fabrication of a single nanogenerator system that can harvest multitype energy remains a challenge.

Among several materials, which are applied as harvesting energy, zinc oxide (ZnO) received great attention due to its unique properties [22–25]. In addition, ZnO has been widely used in sensor [26, 27], solar cells [28–30], and spintronics [31–33] applications. ZnO is a semiconductor material that has a band gap of ~3.3 eV with large excitation binding energy 60 meV and high electron mobility up to 5000 cm^2^/Vs [34]. ZnO has high stability at room temperature with hexagonal wurtzite structure (No. 186, *P*6_3_*mc*) [34]. This structure has a noncentrosymmetric atomic structure, which shows a high polarization [35, 36]. Here, a small change of displacement can be converted into electrical energy.

In this study, we report on morphological modification and characterization technique on ZnO NR. Here, the influence of morphological modification on the behavior of optical absorption and polarization of the ZnO NR is comprehensively studied by introducing different annealing temperatures. This study will give an important key to develop the high nanogenerator device, which can harvest multitype energy.

## 2. Experimental Methods

The vertically aligned ZnO NR on ITO-coated glass substrate have been successfully grown by introducing seed layer on hydrothermal method. The seed layer was prepared from the mixture of zinc acetate dihydrate and ethanol. The seed layer acts as the initial growth for nanorod, which controls the distribution and orientation of ZnO NR. Firstly, the zinc acetate dihydrate and ethanol were stirred at a temperature of 70°C for 45 min to obtain a homogenous solution. Here, monoethanolamine (MEA) was added to the solution and was stirred at a temperature of 70°C for 2 hrs until the solution has a clear and homogenous color. Furthermore, the solution was kept at room temperature for 24 hrs. The seed layer was prepared by the solution using a spin coating method. The fabrication of the seed layer was performed for 25 s with spin that was maintained at 3000 rpm. Moreover, preheating at a temperature of 150°C for 10 min and annealing at 550°C for 2 hrs were performed.

**Figure 1:**
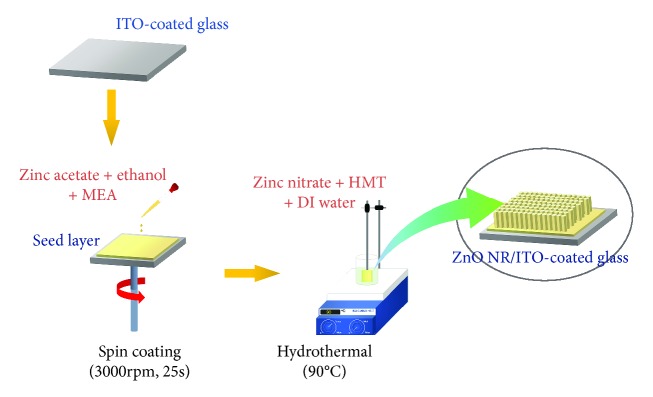
Schematic representation of synthesis of ZnO NR. A seed layer was firstly prepared by spin coating method using zinc acetate, ethanol, and MEA solution.

The ZnO NR were fabricated by using the 45 mM precursor, a mixture of zinc nitrate tetrahydrate, hexamethylenetetramine (HMT), and deionized water. The hydrothermal process was maintained at a temperature of 90°C for 4 hrs. Detail of synthesis of ZnO NR is presented in Figure 1. Furthermore, the morphological modification was performed by introducing different annealing temperatures. Previously, temperature-dependent morphological modification models have been reported [37]. Here, the morphological modification was categorized in several zones according to *T*/*T*_m_, with *T* and *T*_m_ represent applied annealing temperature and melting point (*T*_m−ZnO_ = 1975°C). In this study, we focused on the three types of ZnO NR: without annealing/as-grown ZnO NR (ag-ZnO) and annealed ZnO NR at a temperature of 400°C (ZnO 400°C) and 500°C (ZnO 500°C) for 15 min. Both ZnO 400°C (*T*/*T*_m_ = 0.2) and ZnO 500°C (*T*/*T*_m_ = 0.25) are included in zone *T*, which has a cone-like structure. In this zone, atoms have low activation energy and imply a low surface diffusion. At the applied annealing temperature, the crystals grow out of the initial nucleation and proceed to the top. Here, the materials have a V-shaped columnar crystal with tapered bottoms and domed tops (cone-like), which are separated by voided boundaries [37].

The morphological modification, including distribution and thickness of ZnO NR, was studied by surface and cross-sectional images using scanning electron microscopy (SEM) FEI: INSPECT S50. The surface density was determined by using a surface density model without any additional characterization instrument (e.g., Brunauer-Emmett-Teller (BET) measurement). Furthermore, photoluminescence (PL) spectra were used to confirm structural modification of the samples. The characteristics of optical absorption and polarization of the ZnO NR were investigated by spectroscopic ellipsometry Micropack: Spec-EI 2000.

## 3. Results and Discussion

**Figure 2:**
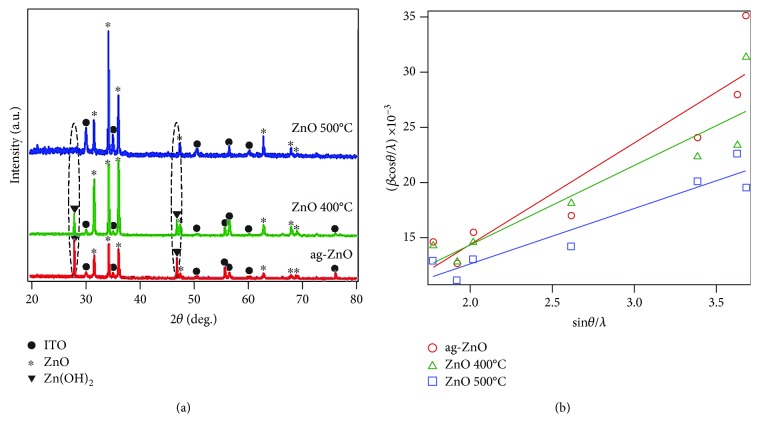
Crystal structure analysis of the ZnO NR on ITO-coated glass grown by hydrothermal method. (a) Diffraction pattern of the ZnO NR on ITO-coated glass. (b) Structural analysis of the ZnO NR on ITO-coated glass by using Williamson-Hall plot. The observed Zn(OH)_2_ phase indicates an incomplete formation of the ZnO NR.

Figure 2 presents the diffraction pattern of the ZnO NR on ITO-coated glass substrate with different annealing temperatures. A hexagonal wurtzite ZnO was successfully obtained by the hydrothermal method. Here, the Zn(OH)_2_ phase (JCPDS No. 00-041-1359) was observed at the diffraction angle of 28° and 47° on the ag-ZnO. This result indicates an incomplete conversion of Zn(OH)_2_ into ZnO. We reveal that annealing leads to the conversion of Zn(OH)_2_ into ZnO, as indicated by the decrease of ZnO(OH)_2_ accompanied by the increase of ZnO peak intensity (JCPDS No. 01-089-7102 and 01-075-1533). In addition, the lattice parameter of the ZnO is obtained by the Rietveld refinement, with the obtained *a* = *b* and *c* value of 3.250 Å and 5.206 Å for ag-ZnO, 3.249 Å and 5.204 Å for ZnO 400°C, and 3.254 Å and 5.212 Å for ZnO 500°C. Further analysis has been performed to determine the structural modification in the ZnO NR. Here, grain size (*D*) and lattice strain (*ε*) of the ZnO NR are determined by using the Williamson-Hall (W-H) plot method as follows:

(1) $$\begin{array}{c} \beta \cos\theta =\frac{K\cdot \lambda }{D}+\varepsilon \sin\theta , \end{array}$$

with *β* is the full width at half maximum (FWHM) of the diffraction peak, *θ* is the diffraction pattern, and *K* is the constant (0.9). The samples have the grain size of 73.1 nm, 70.8 nm, and 78.3 nm and the lattice strain of 2.31 × 10^−3^, 1.80 × 10^−3^, and 1.26 × 10^−3^. The decrease of lattice strain indicates that the ZnO system is towards more stable condition and enhanced crystallinity, which is indicated by the increase of ZnO peak intensity. This result is consistent with the previous result, where the increase of crystallinity of the ZnO NR is attributed to complete conversion Zn(OH)_2_ into ZnO assisted by the higher annealing temperature.

**Figure 3:**
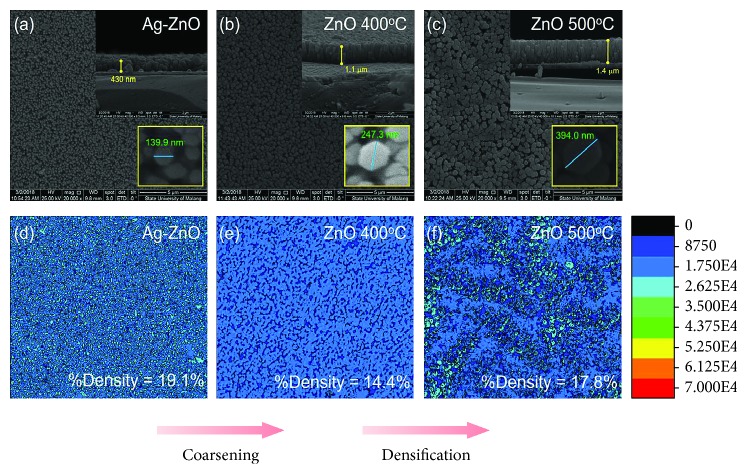
Morphology of the ZnO NR with different annealing temperatures. SEM images of (a) ag-ZnO, (b) ZnO 400°C, and (c) ZnO 500°C and surface density model of (d) ag-ZnO, (e) ZnO 400°C, and (f) ZnO 500°C.

Figures 3(a)–3(c) show the morphological modification of the ZnO NR on ITO-coated glass substrate. Both of the surface and cross-sectional images provided the representation of distribution and the thickness of the samples. The result shows that the use of a seed layer has successfully generated the vertically aligned as-grown ZnO (ag-ZnO), with the thickness of the samples is represented by the length of the nanorod. We found that length and diameter of the ZnO NR increase with applying the higher annealing temperature, with the length of 430 nm, 1.1 *μ*m, and 1.4 μm and diameter of 139.9 nm, 247.3 nm, and 394.0 nm for the ag-ZnO, ZnO 400°C and ZnO 500°C, respectively. In addition, annealing treatment also promotes morphological evolution in ZnO NR. A cone-like shape was successfully obtained by applying annealing treatment at a temperature of 400°C (*T*/*T*_m_ = 0.2) and 500°C (*T*/*T*_m_ = 0.25). In order to investigate the morphological modification of the samples comprehensively, a surface density calculation has been performed. Here, the surface density is determined by using surface mapping from SEM images without additional characterization instrument.

**Figure 4:**
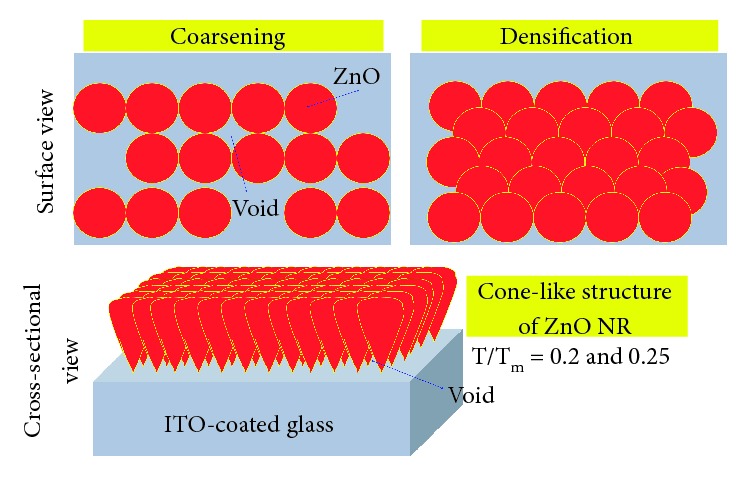
Schematic illustration of distribution of ZnO NR with domination of coarsening and densification. Cone-like structure of ZnO NR provides some void due to shape difference of top and bottom of ZnO NR.

Figures 3(d)–3(f) represent the surface density model of the samples obtained from SEM images. We found that annealing at 400°C is dominated by coarsening of the ZnO sample. Here, the increase in diameter was accompanied by the decrease of the percentage of surface density from 19.21% (ag-ZnO) to become 14.4% (ZnO 400°C). We note that the ZnO 500°C has the denser surface density than that of the ZnO 400°C, with the percentage of surface density of 17.8%. The increase of both diameter and density indicates the domination of densification during annealing at 500°C. Material distributions with coarsening and densification process are illustrated in Figure 4. We infer that the variation of density is originated from domination between coarsening and densification. Both coarsening and densification play a role in distribution rearrangement, which decreases (coarsening) or increases (densification) the density of ZnO NR. In addition, a shape difference of cone-like structure (domed top and tapered bottom sides) provided some void (see Figure 4), which allows light to pass through the ZnO NR and subsequently yield high optical absorption in ZnO NR.

**Figure 5:**
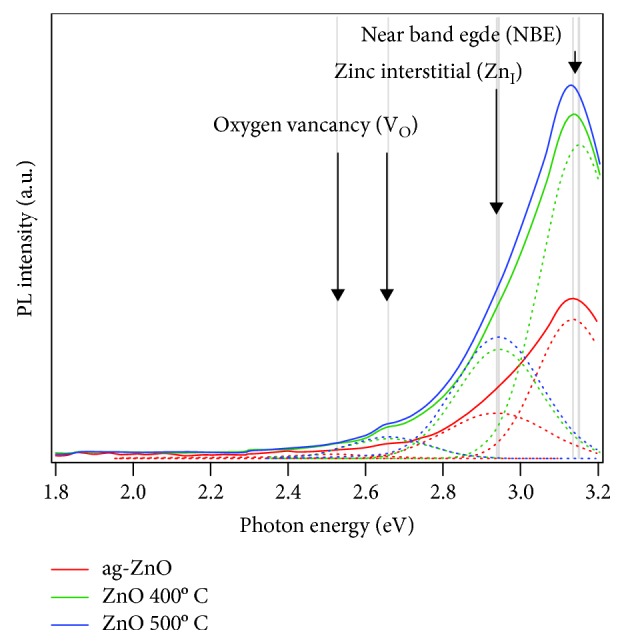
Photoluminescence spectra of the ZnO NR with different annealing temperatures.

Furthermore, the optical characterization was performed to investigate the structural modification and optical properties of ZnO NR.  Figure 5 presents PL spectra of the samples analyzed by multiple Gaussian fitting. We noted that the peaks at 2.53 eV and 2.66 eV are originated from the oxygen vacancy (V_O_). Moreover, the presence of zinc interstitial (Zn_I_) and near band edge (NBE) is observed at 2.941 eV and 3.15 eV. The increase of annealing temperature increases the concentration both of the V_O_ and Zn_I_. Other studies confirmed that the V_O_ and Zn_I_ play an important role in polarization and optical absorption, respectively [36]. Here, the influence of V_O_ and Zn_I_ on optical absorption and polarization of ZnO NR is investigated in detail by spectroscopic ellipsometry.

**Figure 6:**
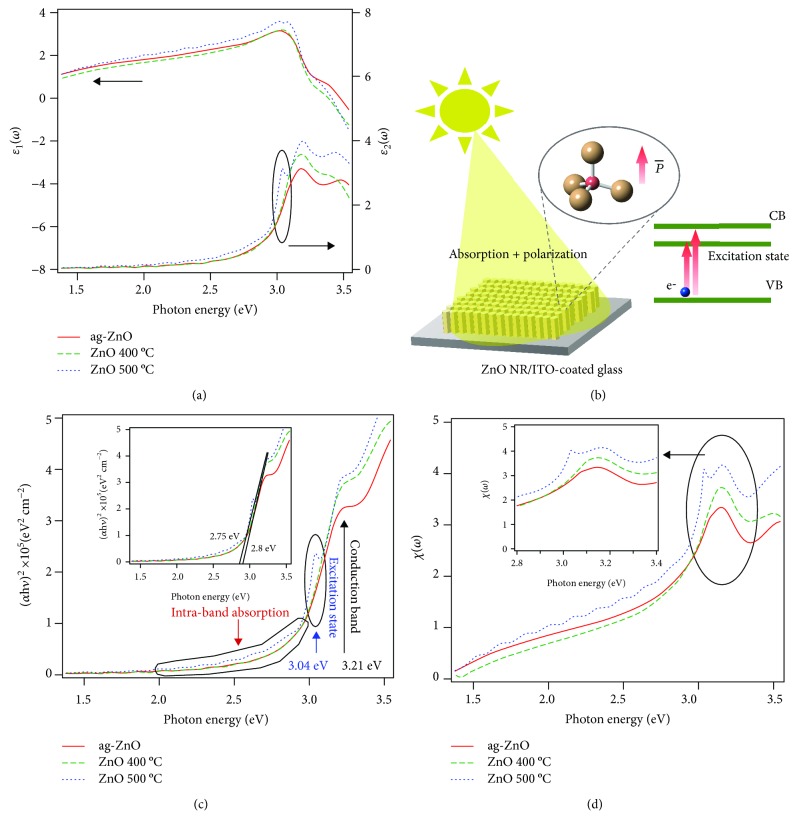
Real and imaginary parts of dielectric function (a), schematic representation of optical absorption and polarization in illuminated ZnO NR (b), absorption coefficient (c), and dielectric susceptibility of ZnO NR (d). A sharp peak at 3.04 eV indicates the excitation state in ZnO 500°C.

Figure 6(a) shows the dielectric function of the ZnO NR with different annealing temperatures. The result confirms that the increase of temperature promotes the increase in both of real (*ε*_1_) and imaginary (*ε*_2_) parts of the dielectric function. Here, the *ε*_1_ and *ε*_2_ are attributed to dielectric polarization and optical absorption of the system, respectively. The result shows that annealing at 400°C promotes the increase of *ε*_2_ (with the maximum intensity observed at an energy of 3.21 eV), while *ε*_1_ shows no significant changes. Moreover, the annealing at 500°C promotes the increase of both *ε*_1_ and *ε*_2_. Intriguingly, a sharp peak of the *ε*_2_ is also obtained by annealing at a temperature of 500°C. These results are potentially due to the morphological modification in ZnO NR.  Figure 6(b) represents the illustration of optical absorption and polarization in illuminated ZnO NR. The electrons excite from valence band (and create a hole) into the conduction band (and excitation state) due to the light absorption. The excited electron and hole interact with the Coulomb interaction, forming an exciton, and promote the dielectric polarization in the system.  Figure 6(c) presents absorption spectra of the ZnO NR with different annealing temperatures. The maximum absorption is observed at an energy of 3.21 eV, which is indicated conduction band. Moreover, the intraband absorption is observed in the energy range of 2.0–2.5 eV, which is presumably due to the contribution of the V_O_ in the ZnO NR. A strong absorption peak, which is related to excitation state, at 3.04 eV is clearly observed on ZnO NR 500°C. This result is potentially due to the double ionization from the high concentration of Zn_I_ in the system. A notable result is confirmed in ZnO NR 500°C, which shows high optical absorption. We infer that the high optical absorption is associated with morphological modification (cone-like structure). In addition, the ag-ZnO and ZnO 400°C show the same band gap of ~2.8 eV, while the ZnO 500°C show the decrease in the band gap of ~2.75 eV. A plausible explanation of these characteristics is due to the existence of the V_O_ and Zn_I_ excitation state, which further provides the wider energy range absorption of light. Previously, low band gap ZnO was reported in other studies, where optical absorption of the ZnO is presented [38]. The decrease of band gap was promoted by the presence of the midgap state in doped ZnO system [38, 39]. In this study, the presence of the midgap state in our system is mainly associated with the existence of the V_O_ and Zn_I_ excitation state.

**Figure 7:**
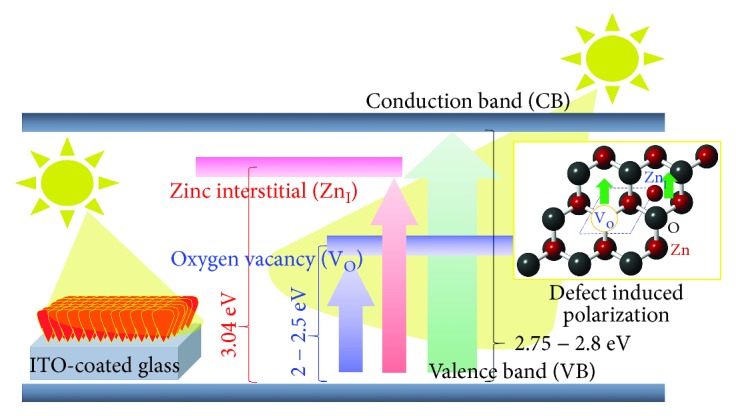
Schematic diagram of the influence of V_O_ and Zn_I_ on optical absorption and polarization of ZnO NR. The existence of both V_O_ and Zn_I_ promoted large number of optical absorption and polarizability of the ZnO NR during light illumination.

The next analysis is focused on the investigation of the polarization of the ZnO NR. The polarization response of the system can be indicated by its susceptibility (*χ*) value. Here, the dielectric susceptibility is obtained from a dielectric function of the ZnO NR by the following relation [35].

(2) $$\begin{array}{c} \chi \left(\omega \right)=\chi^{\prime }\left(\omega \right)+\chi^{\prime \prime }\left(\omega \right), \end{array}$$

(3) $$\begin{array}{c} \chi \left(\omega \right)=\left[\varepsilon_{1}\left(\omega \right)-1\right]-i\varepsilon_{2}\left(\omega \right), \end{array}$$

with *χ*′(*ω*) and *χ*^″^(*ω*) are the real part and imaginary part of dielectric susceptibility.  Figure 6(d) confirms that the dielectric susceptibility of the ZnO NR increases for the higher photon energy in the range of 1.4–3.0 eV and increases significantly at a photon energy of 3.21 eV. Moreover, the same profile is also observed in dielectric susceptibility. The new excitation state promotes a sharp dielectric susceptibility at an energy of 3.04 eV on ZnO 500°C, promoting a wide dielectric susceptibility at energy 3.04–3.21 eV as presented by the blue dashed line in inset of Figure 6(d). The higher and wider dielectric susceptibility provide evidence of the enhancement of polarization in the ZnO NR. The mechanism of optical absorption and polarization of the sample is illustrated in Figure 7. The presence of the V_O_ and Zn_I_ potentially reduce the binding of the system and further promote high polarizability. Moreover, it allows the high polarizability. These results show that the increase of Zn_I_ and V_O_ concentration in the ZnO NR has successfully enhanced their optical absorption and dielectric susceptibility.

## 4. Conclusions

The structure and morphology of the ZnO NR have been successfully modified by introducing different annealing temperatures. The increase of annealing temperature promoted the complete formation of the ZnO phase and the increase of the ZnO NR crystallinity with the more stable condition, as indicated by decreasing in lattice strain. The length and diameter increase with a respective value up to 1.4 *μ*m and 394.0 nm for the annealing at 500°C. Moreover, the change of surface density and increase of oxygen vacancy (V_O_) and zinc interstitial (Zn_I_) concentrations of the ZnO NR were observed at the higher annealing temperature. The optical analysis revealed that the presence of high Zn_I_ and V_O_ concentration changed the band gap from 2.8 to become 2.75 eV and promoted the increase of optical absorption and polarization of the ZnO NR, respectively. Interestingly, the high concentration also created the new excitation state, which enhanced both optical absorption and polarization of the ZnO NR. The coexistence of high optical absorption and polarization will provide a favorable condition which is essential to design ZnO NR-based multitype energy nanogenerators.

## Data Availability

The data used to support the findings of this study are available from the corresponding author upon request.
